# Cross-Sectional Survey of Prophylactic and Metaphylactic Antimicrobial Use in Layer Poultry Farming in Cameroon: A Quantitative Pilot Study

**DOI:** 10.3389/fvets.2022.646484

**Published:** 2022-04-18

**Authors:** Mohamed Moctar Mouliom Mouiche, Frank Dupleix Khalen Wouembe, Serge Eugene Mpouam, Frédéric Moffo, Michael Djuntu, Claude Michel Wombou Toukam, Jean Marc Feussom Kameni, Ndode Herman Okah-Nnane, Julius Awah-Ndukum

**Affiliations:** ^1^Department of Pharmacy, Pharmacology and Toxicology, School of Veterinary Medicine and Sciences, University of Ngaoundéré, Ngaoundéré, Cameroon; ^2^Institute of Agricultural Research for Development, Yaoundé, Cameroon; ^3^National Centre for Animal Husbandry and Veterinary Training, Foumban, Cameroon; ^4^Ministry of Livestock, Fisheries and Animal Industries (MINEPIA), Yaoundé, Cameroon; ^5^Epidemiology-Public Health-Veterinary Association (ESPV), Yaoundé, Cameroon; ^6^College of Technology, University of Bamenda, Bamenda, Cameroon

**Keywords:** antimicrobial use, layer poultry farms, Cameroon, antimicrobial resistance, critically important antimicrobials

## Abstract

An evaluation of the patterns of antimicrobial use in livestock can help understand the increasing level of antimicrobial resistance worldwide. This study aimed at evaluating antimicrobial usage in modern layer poultry farms in the West Region of Cameroon. In this light, 70 layer poultry farms and 4 veterinary pharmacies were surveyed. Data on antimicrobial use were collected through interviews using a quantitative-frequency questionnaire and consultation of medical records. The four veterinary pharmacies sold a total of 2.8 tons of antimicrobials (active ingredients) during 2011. At the level of farms, 297 kg of antimicrobials (active ingredients) were used in the 50 layer poultry farms surveyed. Tetracycline, sulfonamides, quinolones, and β-lactams (aminopenicillins) were the most sold and used drugs in layer farms. As for treatment indication, metaphylactic (58.1%) and prophylactic (41.9%) treatments were the most observed practices, and nearly all (99%) treatments were administered *per os* as remedies to respiratory (33.4%) and digestive (24.7%) tract symptoms. Overall, 78.2% of antimicrobials sold in pharmacies and 67.3% used in the farms belonged to the class of critically important antimicrobials of the WHO categorization of antimicrobials according to their importance to human medicine. Doxycycline, sulfonamide, ampicillin, and streptomycin, which have been banned for layer poultry in the production of eggs for human consumption, were still used in Cameroon. The treatment incidences based on the used daily dose (TI_UDD_) and animal daily dose (TI_ADD_) were 11.59 and 10.45, respectively. In regard to dosage correctness based on the UDD/ADD ratio, aminoglycosides (100%), macrolides (90.6%), and tetracyclines (74.5%) were the most underdosed, while trimethoprim sulfonamides (45.8%) and β-lactams (35.7%) were overdosed. This study highlights an irrational antimicrobial usage in layer poultry farms. Regulation of the use of antimicrobials and the education of farmers on adequate antimicrobial use are essential to preserve the effectiveness of drugs in both humans and animals.

## Background

Intensification of poultry production systems constitutes one of the important challenges of food security in Africa. In the last decades, poultry production systems have been subsequently intensified in response to increased human demand for animal protein intake. In Cameroon, poultry production accounts for at least 34.26% of the total meat harvested from the terrestrial food-producing animals per year, with an estimated headcount of 52 million broilers and layer hens (1). As for egg production, an estimation of 63,382 tons was reported in 2012 to attain 84,129 tons in 2016 progressively (2). Poultry meat and eggs (17 g/day per adult equivalent) represented the second most consumed food of animal origin after fish, estimated at 52 g/day per adult equivalent (3). However, this quantity remains insufficient to satisfy the increasing demand nationwide due to related limited financial resources of livestock farmers and several endemic diseases. Fearing endemic diseases that often reduce productivity, farmers regularly use antimicrobials for disease prevention and control (4). Continuous use of antimicrobials as growth promoters is rampant in Cameroon. The use of antimicrobials results in the presence of residues in food of animal origin and the emergence of antimicrobial resistance (3, 5). Previous studies reported a high prevalence of drug residues of 17% in eggs (6) and 48% in poultry meat (7) and high pooled prevalence of multidrug-resistant *Escherichia coli* isolates in humans (47%) and animals (76%) to all classes of antimicrobials (including fluoroquinolones, carbapenems, and third-generation cephalosporins) used for treatment in communities, hospital settings, and animal health centers in Cameroon (5, 8). Insufficient application of biosecurity measures in farms and a lack of awareness of farmers coupled with the lack of veterinary oversight on antimicrobial use constitute the driving factors of inappropriate antibiotic therapy (9, 10). In addition, a lack of strict regulation favors illicit importation, clandestine distribution of drugs, and automedication by farmers, with obvious consequences like the emergence of resistant bacteria (11). Monitoring the use of antimicrobials in food-producing animals is one of the cornerstones for policy- and decision-making in the fight against antimicrobial resistance. Quantitative data on antimicrobial use are very scarce in sub-Saharan African countries. Antimicrobials are commonly used in livestock for prophylactic, metaphylactic, and therapeutic purposes. Prophylactic use of antimicrobials is defined as the treatment of healthy animals to prevent diseases from occurring, whereas metaphylactic use is defined as the treatment of clinically healthy animals belonging to the same group as animals that showed clinical symptoms of diseases (12). However, little is known about the quantities of antimicrobials administered and the frequency of their use in livestock. Specifically, this study aimed at evaluating antimicrobial therapy practices and the quantities of antimicrobials used in modern layer poultry farms in the West Region of Cameroon.

## Methods

### Study Site and Study Design

**Figure 1:**
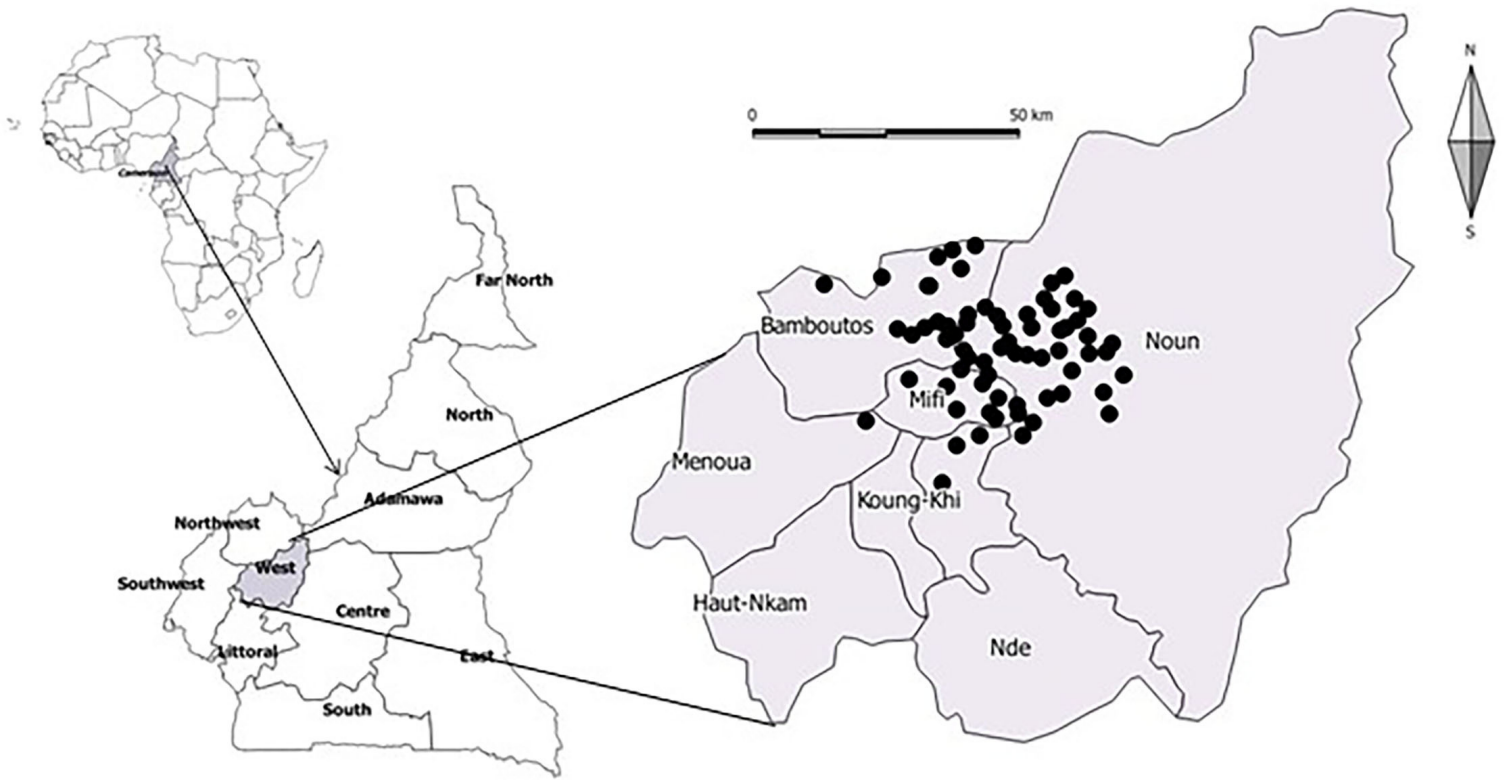
Map showing study areas in the West Region of Cameroon.

This cross-sectional survey was conducted from July 2012 to March 2013 in the West Region (5°25′0″-5°35′0″N−10°20′0″-10°35′0″E) of Cameroon, located in the Equato-Guinean highland, with an average annual rainfall of 1.600–2.000 mm. The rainy season stretches from mid-March to December, with temperatures ranging between 23 and 25°C (Figure 1). Poultry production in this region accounts for 36.8% of the national population of domestic poultry (broiler and layer chicken), and egg production represents 82.3% of the national production per year (2). During data collection, a total of 11 veterinary pharmacies were identified in the study area, four of which were willing to participate in the study and agreed to provide quantitative information. At the farm level, a minimum sample size of 62 poultry farms was estimated based on previous reports on the level of antimicrobial misuse of 82% (3) with a confidence interval of 95% and precision of 10%. The random number generation technique was used to select farms from a list of poultry farmers available at the West Regional Delegation of the Ministry of Livestock, Fisheries, and Animal Industries. During the investigation, 70 non-integrated farms (that got hens from elsewhere) with a minimum capacity of 1,000 chickens per farm were included in the study. Participation in the study was voluntary, and no incentive was given to the participating farmers. At the level of veterinary pharmacies, data on the quantities of drugs sold during 2011 were collected using treatment records provided by the pharmacists. In each poultry farm, information on antimicrobial use obtained from farm records included the following: product administered, amount administered, dosage and duration of administration, administration route, product category, diagnosis, indication (preventive treatment or metaphylactic treatment) number, and age of treated birds. The quantity of antimicrobials used by farmers was collected for one production cycle in all stables of the farms.

### Data Analysis

#### Antimicrobials Sold by Pharmacies

The collected data were entered in an Excel spreadsheet (Microsoft Corporation, Redmond, WA, USA). The total amount by weight of each antimicrobial active ingredient per drug (kilogram), converted to tons, was obtained by multiplying the quantitative composition of the active ingredient for each pharmaceutical form by the number of units sold. For some active ingredients expressed in international unit, a conversion factor was applied to calculate the amount of antimicrobial ingredient following OIE recommendations (13, 14). To evaluate the impact of veterinary antimicrobial use on public health, the antibiotics were classified according to the WHO categorization of antimicrobials according to their importance in human medicine (15).

#### Quantification of Antimicrobial Consumption in Layer Poultry Farms

Volumes of antimicrobials administered for prophylactic and metaphylactic treatments were converted to milligrams of active substance per kilogram of live weight. The frequency of use of different active ingredients was calculated. Drug quantification was done using weight indicators. As described by Persoons et al. (16), the animal daily dose (ADD), which is the assumed average dose per day and per kilogram of chicken of a specific drug, was collected from the drug's instruction leaflet.

The used daily dose (UDD), which describes the amount of active substance actually administered to the animals in mg/kg, was calculated based on the following formula (17, 18):


$$\begin{array}{l} Used\;Daily\;Dose\;\left(mg/kg\right)\\ =\frac{\text{Amount of antimicrobials }\left(\text{active substance}\right)\text{ adminitered }\left(\text{mg}\right)}{\text{Number of chiken treated }\times \text{Mean standard weight }\left(\text{kg}\right)\times \text{treatment days }} \end{array}$$

The UDD/ADD ratios were calculated to assess the correctness of dosage. Ratios between 0.8 and 1.2 inclusive were considered as correct dosage (12). Values <0.8 and >1.2 were considered as underdose and overdose, respectively.

The frequency of treatments was quantified by calculating treatment incidences (TIs) (19). This TI based on the ADD gives TI as it should be when the prescribed dose is applied, or based on the UDD, this gives the TI as it is in reality. The following formula was used to calculate TIs:


$$\begin{array}{l} \frac{Total\;amount\;of\;antimicrobial\;administered\;\left(mg\right)}{UDD\;or\;ADD\;\left(mg/kg\right)\times number\;of\;days\;at\;risk\times kg\;chicken\;at\;risk} \end{array}$$

In this equation, the total amount of antimicrobial administered is calculated per compound. The number of days at risk is the time—in days—a layer is possibly exposed to one or more treatments. This was estimated to be 356 days, the minimum time during which layer farmers keep laying hens. The kg of a chicken was calculated as the number of chickens multiplied by their mean weight. This weight at treatment was standardized for the different flocks by dividing the sum of the total weight of birds at each treatment instance by the number of birds multiplied by the number of treatments (17). The treatment incidence for chickens is thus defined as the number of chickens per 1,000 that are treated daily with one ADD or UDD. To determine whether significant differences exist between TI_UDD_ and TI_ADD_, a paired sample *t*-test was used. The assessment of potential risk factors influencing the use of antimicrobials was done using a multivariable logistic regression model. The odds ratio was used to examine the degree of association, the confidence interval was set at 95%, and the significance was at *p* < 0.05. All data were computed using IMB Statistics software (SPSS Inc., Chicago, IL, USA).

## Results

### Demographic and Farm Characteristics of Surveyed Poultry Farms in the West Region of Cameroon

Of the 70 farmers surveyed in the study area, 56 (80%) had at least a primary level of education and 14 (20%) had no formal education. Only 10 (14.3%) had received training in poultry farming and 32 (45.7%) had 5–10 years of experience in poultry farming.

Less than half (31/70, 44.3%) of farms surveyed had flock sizes between 5,000 and 10,000. Almost 36 (51.4%) had a stocking density of 7–8 hens/m^2^. More than half (39/70, 55.7%) of the farms surveyed had a livestock ratio of 2,000–3,000 chickens per worker (Table 1). Regarding hygiene management of the surveyed poultry farms, it was observed that only 8.60% (6/70) respected the recommended minimum distance of 500 m between two farms and more than 54.3% (38/70) met the construction standards of poultries with concrete floors and a system limiting the entry of wild birds into the building. Almost 35.7% (25/70) of farms practiced the single-band system (all-in-all-out), while all surveyed farms (100%) recycled packaging materials such as egg trays and cartons. In almost 51.4% (36/70) of farms, dead chickens were served for human consumption, and in 11.4% (8/70), dead chickens were incinerated or buried. As for frequently occurring diseases, infectious bronchitis virus infection, mycoplasmosis, and pasteurellosis were mostly reported in the dry season, while salmonellosis, colibacillosis, and coccidiosis were commonly reported in the rainy season.

**Table 1:** Characteristics of 70 poultry farms surveyed.

Poultry herd size (hens)	<5,000	5,000–10,000	>10,000
Percentage (%)	18.6	44.30	37.1
Density during the laying phase (hens/m^2^)	≤ 7	7–8	>8
Percentage (%)	34.3	51.4	14.3
Livestock ratio peremployee	<2,000	2,000–3,000	>3,000
Percentage (%)	18.6	55.70	25.70

### Quantities of Antimicrobials Sold by Veterinary Pharmacies by Weight of Active Substances

**Figure 2:**
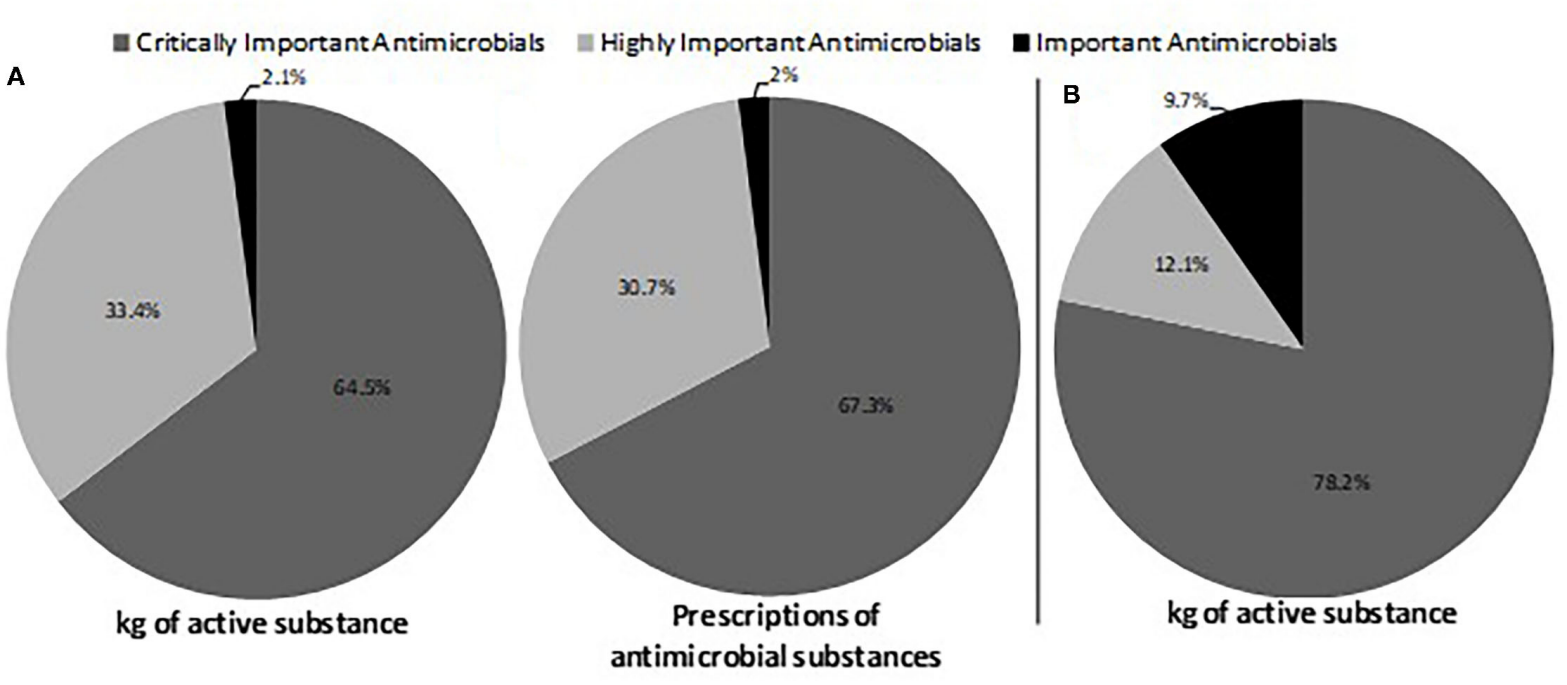
Classification of the antimicrobials used according to the WHO categorization of antimicrobials according to their importance to human medicine. **(A)** Percentages of the amount of active substance in each category for four veterinary pharmacies in the West Region of Cameroon in 2011. **(B)** Percentages of the amount of active substance and percentages of prescriptions in each category for 50 layer poultry farms in the West Region in Cameroon from January 2012 to December 2012.

The four veterinary pharmacies surveyed in the West Region of Cameroon sold a total amount of 2.8 tons (Table 2) of antimicrobials (active ingredients) in 2011. The investigation shows that mainly eight antimicrobial classes were sold, with the highest being tetracyclines (62.1%), followed by quinolones (11%) and sulfonamides (10%). The least dispensed antimicrobials were macrolides (1.6%) and aminoglycosides (1%). Almost 78.2% of the total antimicrobial sale corresponded to the WHO categorization of antimicrobials according to their importance to human medicine (Figure 2A).

**Table 2:** Percentage of the total amount of most important antimicrobial classes sold in four (*n* = 4) veterinary pharmacies in the West Region of Cameroon in 2011.

**ATCvet**	**Active substance**	**Percentage of the active substance**				
		**VP1**	**VP2**	**VP3**	**VP4**	**Total**
QJ01G	Aminoglycosides	1.2	9.8		5.4	1.0
QJ01E	Sulfonamides–trimethoprim	7.8		11.4	18.6	10.0
QJ01F	Macrolides	2.3		0.6	3.9	1.6
QJ01XB	Nitrofurans		2.2	25.8	7.1	9.8
QJ01XE	Polymyxins	2.0	3.7	1.5	2.7	2.0
QJ01M	Quinolones	14.4	30.6	2.8	4.3	11.0
QJ01A	Tetracyclines	68.7	53.7	56.2	56.1	62.1
QJ01C	β-Lactams	3.5		1.7	2.0	2.5

*A total amount of 2.8 tons of active substance was included in the analysis*.

*VP, Veterinary pharmacy; ATCvet, Anatomical Therapeutic Chemical Classification System for veterinary medicinal products*.

### Evaluation of Antimicrobials Used in Poultry Farms

#### Qualitative Estimate of Antimicrobial Usage in Layer Farms in the West Region of Cameroon

Of the 70 farms investigated, 50 (71.43%) kept records of antimicrobial use, while 20 (28.57%) without the antimicrobial treatment records were excluded from the analysis. Analysis of the records indicated 667 antimicrobial prescriptions (Table 3). Healthcare professionals intervened six to seven times in disease cases/issues per production cycle. Antibiotic treatment failures were experienced two to three times per barn. In the case of treatment failure, 28% of health workers increased the dose and prolonged the treatment duration. In the case of respiratory or digestive tract infection, 14.3% of health workers chose to extend the treatment duration, while 57.1% prescribed another antimicrobial or a combination of antimicrobials known to be efficacious against the pathogens. It was also observed that 33% of farmers regularly practiced automedication. Almost 42% of farmers obtained antimicrobials from veterinary pharmacies, while 54% and 4% purchased from local vendors and health workers, respectively, through an unofficial channel. Antimicrobials were mostly administered via drinking water (98.6%) by the farmers, while 1.4% of injected drugs were administered by a health worker.

**Table 3:** Consumption of antimicrobials (in amount) and the number of prescriptions from 50 layer poultry farms in the West Region in Cameroon from January 2012 to December 2012.

**ATCvet**	**Active compound**	**Amount (kg)**	**Percentage**	**Number of prescription**	**Percentage**
QJ01G	Aminoglycosides	3.9	1.3	63	9.3
QJ01E	Sulfonamides–trimethoprim	78.6	26.4	96	14.2
QJ01F	Macrolides	9.1	3.1	32	4.7
QJ01XB	Nitrofurans	6	2.0	14	2.1
QJ01XE	Polymyxins	12.8	4.3	130	19.2
QJ01M	Quinolones	35.7	12.0	83	12.3
QJ01A	Tetracyclines	130.6	43.9	231	34.1
QJ01C	β-lactams	20.7	7.0	28	4.1
Total		297.4		677.0	

*A total of 677 treatments (297 kg of active substance) were included in the analysis*.

*Prescription of combinations of antimicrobials from different classes is counted as one prescription for each component ingredient*.

*ATCvet, Anatomical Therapeutic Chemical Classification System for veterinary medicinal products*.

#### Quantitative Estimate of Antimicrobial Usage in Layer Farms in the West Region of Cameroon

From the 50 poultry farms that had tracking records of antimicrobial use, 371,696 chickens with a total biomass of 408,865.6 kg were recorded. In total, 297 kg of antimicrobials (active ingredients) was recorded during one production cycle in all of the investigated farms. However, a mean quantity of 726.40 mg of antimicrobials (active ingredients) per kilogram of chicken biomass was obtained. Antimicrobials of the tetracycline class were the most used (43.9%), followed by sulfonamides (26.4%) and quinolones (12%) (Table 3). Almost 67.3% of the total antimicrobial use corresponded to the WHO categorization of antimicrobials according to their importance in human medicine (Figure 2B).

**Figure 3:**
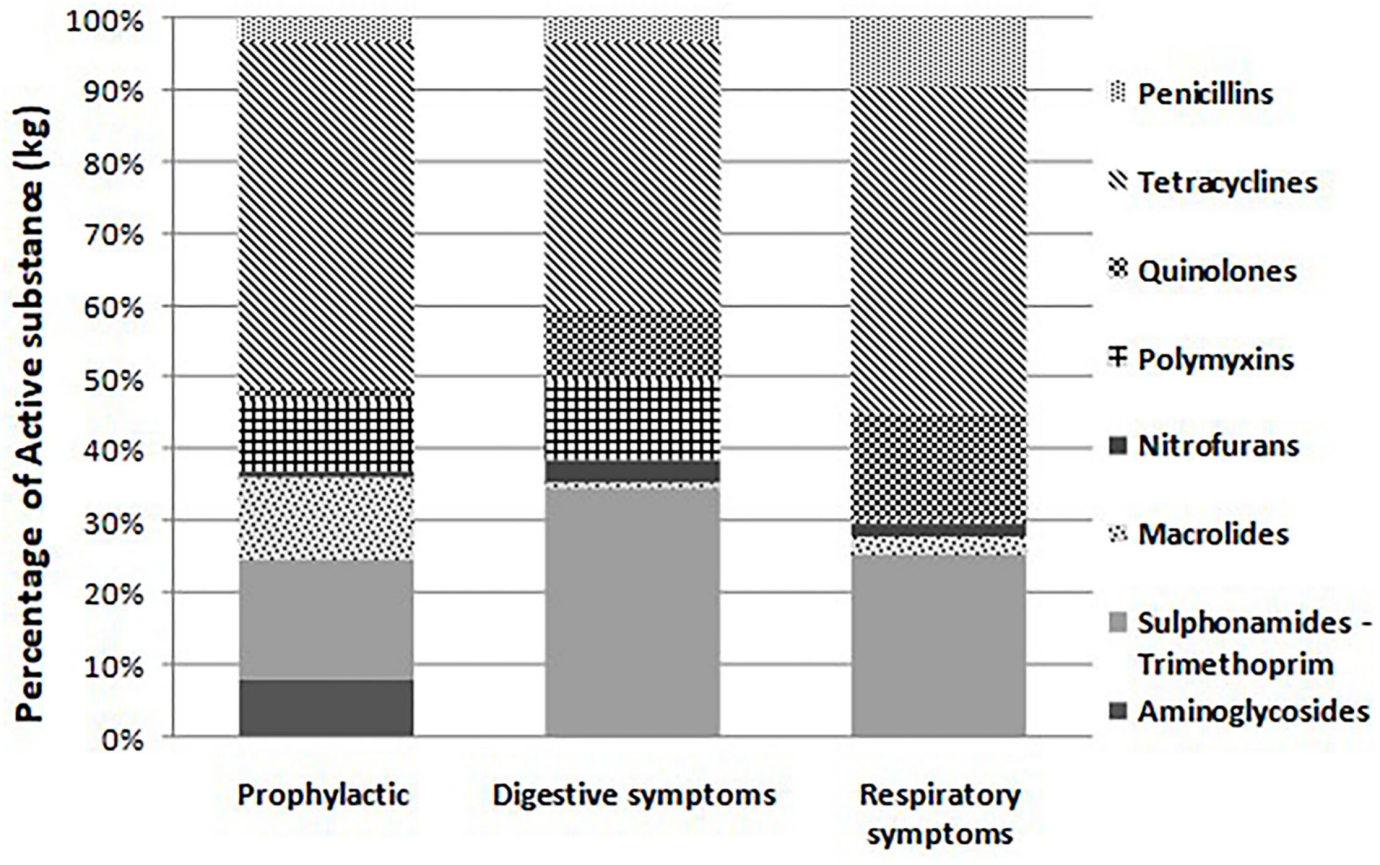
Distribution of different antimicrobial classes used in 50 layer poultry farms in the West Region of Cameroon according to the different indications of use. Of the 297 kg of antimicrobials used, 11.8% are for prophylactic use and 88.2% for metaphylactic use (24.3% for digestive symptoms and 63.9% for respiratory symptoms).

Of 297 kg of antimicrobials recorded, 11.8 and 88.2% were used for prophylactic and metaphylactic treatments, while 24.3 and 63.9% were used against digestive and respiratory tract disorders, respectively (Figure 3). Quinolones were the least used for prevention, while macrolides and nitrofurans were the least used for metaphylactic therapy of gastrointestinal and respiratory symptoms, respectively. The average dosage applied in farms, described as the ADD and UDD, is summarized in Table 4. From the UDD/ADD ratio, it was observed that aminoglycosides and macrolides were usually underdosed, while trimethoprim, sulfonamides, and β-lactams (aminopenicillins) were slightly overdosed. Nitrofurans and quinolones were usually administered within the proper dose range. According to indications, antimicrobials were underdosed during prophylactic treatment (Table 4). Independent of the active substances, indications, and administration routes, the average of TI_UDD_ was 11.59, which implies that, on average, 12 out of 1,000 chickens were treated daily with a UDD. This average was greater than that of TI_ADD_, but the difference observed was not significant (*p* > 0.05), which means that, at equal doses, more chickens were treated with a UDD compared to an ADD (Table 5). The most used antimicrobial agent was oxytetracycline, followed by colistin and the combination of sulfonamides and trimethoprim, while the least used were tetracycline and the combination of benzylpenicillin procaine and spiramycin–trimethoprim.

**Table 4:** Correctness of dosage (UDD/ADD ratio) according to the different antimicrobial classes administered and different indications of use in layer farms in the West Region in Cameroon between January and December 2012.

		**Correct dosing (%) 0.8 < UDD/ADD >1.2**	**Underdosing (%) UDD/ADD <0.8**	**Overdosing (%) UDD/ADD > 1.2**
Antimicrobial class	Aminoglycosides		63 (100)	
	Sulfonamides–trimethoprim	14 (14.6)	38 (39.6)	44 (45.8)
	Macrolides	1 (3.1)	29 (90.6)	2 (6.3)
	Nitrofurans	7 (50)	5 (35.7)	2 (14.3)
	Polymyxins	13 (10)	82 (63.1)	35 (26.9)
	Quinolones	14 (16.9)	53 (63.9)	16 (19.3)
	Tetracyclines	13 (5.6)	172 (74.5)	46 (19.9
	β-lactams		18 (64.3	10 (35.7)
Indications	Prophylactic	15 (4.5)	295 (89.4)	20 (6.1%)
	Metaphylactic/digestive symptoms	20 (14.4)	70 (50.4%)	49 (35.3)
	Metaphylactic/respiratory symptoms	27 (12.9)	95 (45.5)	87 (41.6)
Total		62 (9.3)	460 (68.9)	155 (23.2)

*A total of 677 treatments were included in the analysis*.

*Prescriptions of combinations of antimicrobials from different classes are counted as one prescription for each component ingredient*.

**Table 5:** Distribution of treatment incidence according to the active substances, indications, and administration routes for poultry farming in the West Region of Cameroon between January and December 2012.

**Active substances**	**TI**_**ADD**_				**TI**_**UDD**_			
	**Min**	**Mean**	**Max**	**Overall**	**Min**	**Mean**	**Max**	**Overall**
Amoxicillin	1.65	27.79	95.67	528.00	2.74	14.85	35.62	282.19
Ampicillin	0.70	6.06	19.88	42.39	2.74	10.18	13.70	71.23
Ciprofloxacin	11.40	15.96	26.63	79.78	8.22	12.05	19.18	60.27
Colistin sulfate	0.70	13.38	126.16	1,458.59	2.74	11.34	21.92	1,235.62
Colistin and trimethoprim	0.26	8.16	29.19	342.80	2.74	9.65	24.66	405.48
Doxycycline	0.00	20.55	83.00	780.71	5.48	13.91	21.92	528.77
Enrofloxacin	8.42	26.71	97.72	560.96	8.22	15.26	46.58	320.55
Erythromycin	0.03	5.28	47.59	142.43	2.74	12.99	24.66	350.68
Flumequine	0.46	3.21	24.96	70.54	2.74	11.08	16.44	243.84
Furaltadone	1.18	6.72	14.49	9.08	5.48	9.78	13.70	136.99
Neomycin	0.03	0.30	1.82	11.76	2.74	9.20	16.44	358.90
Norfloxacin	1.78	7.74	15.88	270.85	8.22	13.07	19.18	457.53
Oxytetracycline	0.02	6.07	79.89	1,164.75	2.74	10.73	30.14	2,060.27
Procaine benzylpenicillin	0.01	0.01	0.01	0.02	2.74	2.74	2.74	5.48
Spiramycin and trimethoprim	1.23	8.01	14.79	16.02	8.22	8.22	8.22	16.44
Streptomycin	0.04	1.78	5.01	42.72	2.74	11.42	24.66	273.97
Sulfamides and trimethoprim	0.48	19.68	78.14	1,456.59	5.48	13.03	21.92	964.38
Tetracycline	4.48	4.48	4.48	4.48	21.92	21.92	21.92	21.92
Tylosin	0.12	1.92	4.03	7.68	13.70	13.70	13.70	54.79
**Indications**								
Prevention	0.00	3.03	47.59	995.66	2.74	10.20	24.66	3,356.16
Therapeutic	0.01	17.47	126.16	6,079.47	2.74	12.91	46.58	4,493.15
Digestive disorders	0.04	16.78	126.16	2,332.68	2.74	12.52	30.14	1,739.73
Respiratory disorders	0.01	17.93	97.72	3,746.79	2.74	13.17	46.58	2,753.42
**Administration route**								
Oral	0.00	10.54	126.16	7,051.10	2.74	11.63	46.58	7,783.56
Injectable	0.01	3.00	8.86	24.04	2.74	8.22	13.70	65.75
Overall	0.00	10.45^a^	126.16	7,075.14	2.74	11.59^a^	46.58	7,849.32

*TI_ADD_, Treatment incidence based on ADD; TI_UDD_, Treatment incidence based on UDD*.

a *The treatment incidence averages do not differ significantly with the threshold at a confidence interval of 95%*.

#### Risk Factors for the Antimicrobial Dosage Used in Layer Farms in the West Region of Cameroon

The poultry herd size, the density of hens per square meter, and the livestock ratio per employee were observed as risk factors for antimicrobial dosage in poultry farms in the study area. Multivariable logistic regression showed that poultry herd sizes of fewer than 5,000 hens (OR = 0.17, *p* = 0.001) were associated with underdosage of antimicrobials, while poultry herd sizes between 5,000 and 10,000 hens (OR = 0.14, *p* = 0.001) were observed to be significantly associated with overdosage of antimicrobials. Densities <7/m^2^ (OR = 3.2^e−008^, *p* = 0.001) were significantly associated with overdosage of antimicrobials. A livestock ratio of fewer than 2,000 hens per employee (OR = 3.2^e−008^, *p* = 0.001) was significantly associated with overdosage of antimicrobials (Table 6).

**Table 6:** Factors that affected the correct usage of antimicrobials in layer farms in the West Region of Cameroon.

**Factors**	**Correct usage of dosage**	**Variables**	**Number of treatment (%)**	**Odds ratio (95% CI)**	***p*-value**
Poultry herd size	Underdose	<5,000	380 (56.2%)	0.17 (0.059–0.0478)	0.000
		5,000–10,000	288 (42.6%)	0.07 (0025–0.209)	
		>10,000	8 (1.2%)	1.0	
	Overdose	<5,000		0.32 (0.103–0.982)	0.001
		5,000–10,000		0.14 (0.044–0.446)	
		>10,000		1.0	
Stocking density (number of chickens/m^2^)	Underdose	<7	337 (49.9%)	0.085 (0.00–1.18)	1.000
		7–8	178 (26.3%)	1.25 (0.000–2.25)	
		>8	161 (23.8%)	1.0	
	Overdose	<7		3.20^e−008^ (1.803^e−008−^5.693^e−008^)	0.000
		7–8		2.84^e−008^ (2. 838^e−008−^2.838^e−008^)	
		>8		1.0	
Livestock ratio per employee	Underdose	<2,000	380 (56.2%)	0.85 (0.000–1.18)	1.000
		2,000–3,000	288 (42.6%)	1.25 (0.000–2.25)	
		>3,000	8 (1.2%)	1.0	
	Overdose	<2,000		3.20^e−008^ (1.803^e−008−^5.693^e−008^)	0.000
		2,000–3,000		2.84^e−008^ (2.838^e−008−^2.838^e−008^)	
		>3,000		1.0	

## Discussion

The present study was carried out to evaluate antimicrobial therapy practices and antimicrobial quantities used in modern layer poultry farms in the West Region of Cameroon. The findings highlight that more than half of the surveyed layer farmers used antimicrobials from unofficial or illicit channels. The Cameroonian legislation stipulates that veterinarians and licensed human pharmacists in Cameroon can import and distribute veterinary drugs. However, inadequate border controls and a lack of strict regulation favor illicit importation of veterinary drugs by non-health professionals, clandestine distribution, and automedication by farmers. Also, due to the lack of application of drug regulations, farmers can purchase antimicrobials without a veterinary prescription. This contributed to the obvious high level of medication of livestock (11).

This study reveals that antimicrobials were commonly used in modern layer poultry farms in the West Region of Cameroon. The most sold antimicrobials by veterinary pharmacies included in the study in 2011 were tetracyclines, sulfonamides, quinolones, and nitrofurans. In addition, four-fifths of sales corresponded to the WHO categorization of antimicrobials according to their importance in human medicine. This result is similar to those observed by Têko-agbo et al. (20) in Cameroon and Senegal, where tetracyclines were the most sold antimicrobials.

As for the evaluation of antimicrobial use in layer poultry farms, 50 (71.4%) out of the 70 initially selected were investigated. Twenty farms that did not keep records of on-farm antimicrobial use were not included in the analysis. The non-archiving of treatment records by some farmers might be due to their lack of awareness or knowledge on its importance for monitoring antimicrobial use. Those with antimicrobial treatment records were also observed to misuse drugs. These results show that all farmers used one or more antimicrobial drugs for metaphylactic and prophylactic purposes. This is in line with the findings reported in the Center Region of Cameroon (4) and Nigeria (21). Overuse of antimicrobials in poultry farms may be due to a lack of adequate biosecurity measures reported in various studies on Cameroon (10, 22). Also, it is worth noting that intensification of poultry production is linked to high importation, consumption, and widespread use of veterinary drugs in Cameroon (23). Tetracyclines, quinolones, and sulfonamides were the most used antimicrobial classes in layer poultry farms. Similar results were reported for the Center (4) and West (24) Regions of Cameroon. Widespread use of tetracyclines in livestock farming can be explained by their broad spectrum of therapeutic action (25), cheaper cost, and use as growth factors in poultry farming (26). Studies on antimicrobial resistance indicate consistent detection of high resistance levels to tetracyclines. Mouiche et al. (5) reported a pooled prevalence of resistance of *E. coli* to tetracycline (85.5%) and doxycycline (68.2%) in Cameroon. In addition, *E. coli* isolated from poultry litter in Cameroon showed a high prevalence of resistance to tetracycline (79%) and doxycycline (88%) (6).

The use of quinolones—a WHO's Highest Priority Critically Important Antimicrobial class (15)—in the present study could be associated with decreased efficacy of tetracyclines and sulfonamides (4). However, the use of quinolones is of deep concern since these drugs are commonly used to treat multidrug-resistant *Salmonella* spp. infections in humans (27). Besides, the use of quinolones in chicken favors the development of quinolone-resistant Campylobacter, an etiologic agent of gastroenteritis in humans (28). The absence of a prohibited list of antimicrobials for livestock at the national level encourages the importation and use of banned antimicrobials. For example, 2% of nitrofurans was sold and used in layer poultry farms in the West Region of Cameroon. This antibiotic has been banned from being used in food-producing animals since 1991 in the United States and 1995 in the European Union due to concerns over its carcinogenicity (29).

In this study, antimicrobial use estimated based on animal population (adjusted) indicated that each layer received 726.4 mg of active ingredient per kilogram of live weight and at least 10% of this for preventive purposes. The quantity observed was higher than 30.35 mg/kg reported in the OIE Africa region (30), 63.48 mg/kg in Morocco (31), and 265.1 mg/kg in Vietnam (32). Several poultry pathologies are endemic in Cameroon, and biosecurity measures are poorly implemented by farmers, resulting in the overuse of antimicrobials for disease prevention (10). Farmers do not regularly clean up their drinking water canals, which could lead to biofilm formation (bacteria, fungi, minerals, etc.) in pipes (33). This biofilm could decrease solubility of certain antimicrobials, resulting in insoluble complex formations, and a decrease in effectiveness of water treatment by chlorination, the most commonly used water treatment method in Cameroon (4). The administration route observed in this study is in line with that of Kamini et al. (4), who reported that 99% of treatments were done *per os* in the Center Region of Cameroon. The oral route is prioritized for mass medication, through which incorrect dosing in water can increase risks of intoxication from high antibiotic intake compared to parenteral routes. The oral route permits the administration of drugs in solid, semiconsistent, and liquid forms through drinking water or mixing with feed (34). Also, the oral route does not require expertise, favoring high automedication of animals by farmers. The preferred oral administration route observed in the present study explains the higher proportion of powdered pharmaceutical forms imported during the study period.

From the UDD/ADD ratio, it appeared that several antimicrobials were either underdosed or overdosed. The high percentage of underdosed antimicrobial could be explained by the fact that at least 40% of antibiotics were administered for preventive purposes. In this treatment indication, farmers regularly reduce the drug dose. Sublethal antimicrobial concentrations can induce stress in the targeted bacteria, which may favor mutation and might also result in a transient decrease in antimicrobial susceptibility due to increased numbers of resistant bacteria (35). Evidence studies revealed that pig feed with subtherapeutic supplemented tylosin presented a significantly higher level of tylosin-resistant anaerobic bacteria compared to the control group after only 3 weeks with an increased resistance pattern from 11.8 to 89.6% (36). Irrespective of the active substance, indications, and routes of administration, the average treatment incidence, TI_UDD_, was lower than 382.6, as reported by Van Cuong et al. (37) in Vietnam. A lack of hygiene observed in poultry farms in Cameroon increases the risk of disease emergence (10), which would favor a greater use of preventive treatments, resulting in high incidence of treatment in farms (29).

Factors associated with antimicrobial usage in poultry farms in this study comprised stocking density, poultry herd size, and livestock ratio per employee. These observations were in line with Moffo et al. (38), who reported a negative correlation between the stocking density (chickens per square meter) and knowledge on antimicrobial use in poultry farming in Cameroon. Also, poor poultry production management practices provide favorable conditions for the emergence of pathogens and antimicrobial use. Weak biosecurity measures were more often observed in small flock-size poultry farms than large flock-size (39) farms and might explain the misuse of antimicrobials to prevent or control diseases.

Frequent practice of metaphylactic and prophylactic treatments was observed with underdosing of critically important antimicrobials according to their importance in human medicine. Also, the use of some antimicrobials that are prohibited in many countries was observed, including the overdose of some critically important antimicrobials. Given these results, it is therefore incumbent on the regulatory bodies to strengthen the training and awareness of all actors in the poultry sector. Poultry farmers should be encouraged to adopt good biosecurity practices, while using antimicrobials should be rationalized by establishing guidelines for good antibiotic therapy practice. Data used in this study were collected 10 years ago, and this might represent a limitation of the findings in respect of the current antimicrobial use in layers in Cameroon. Meanwhile, this study could inform AMU surveillance in Cameroon and orientate the possible collection of quantitative data. It also provides baseline information for the antimicrobial resistance surveillance program at the national level. Given that, from 2014 to 2019, we observed an increase in antibiotic importation (23), it will be important to re-evaluate the quantities used in layer farms to assess the impact of antimicrobial misuse. However, tools are needed to be refined to collect more accurate farm information for the better understanding of antimicrobial use in livestock in Cameroon.

## Data Availability Statement

The raw data supporting the conclusions of this article will be made available by the authors, without undue reservation.

## Author Contributions

MM and SM conceived, designed, and coordinated the study. FW, CT, and MM coordinated the field work and data collection. MM, FW, FM, and MD carried out the analysis. FM, MM, and NO-N prepared the first draft of the manuscript. JK, NO-N, CT, and JA-N critically reviewed the manuscript. All authors have read and approved the final version of this manuscript.

## Conflict of Interest

The authors declare that the research was conducted in the absence of any commercial or financial relationships that could be construed as a potential conflict ofinterest.

## Publisher's Note

All claims expressed in this article are solely those of the authors and do not necessarily represent those of their affiliated organizations, or those of the publisher, the editors and the reviewers. Any product that may be evaluated in this article, or claim that may be made by its manufacturer, is not guaranteed or endorsed by the publisher.
